# Diversity of *Actinobacteria* Isolated from Date Palms Rhizosphere and Saline Environments: Isolation, Identification and Biological Activity Evaluation

**DOI:** 10.3390/microorganisms8121853

**Published:** 2020-11-25

**Authors:** Omar Messaoudi, Joachim Wink, Mourad Bendahou

**Affiliations:** 1 Laboratory of Applied Microbiology in Food, Biomedical and Environment, Abou Bekr Belkaïd University, 13000 Tlemcen, Algeria; bendahou63@yahoo.fr; 2 Department of Biology, Faculty of Science, University of Amar Telidji, 03000 Laghouat, Algeria; 3 Microbial Strain Collection, Helmholtz Centre for Infection Research GmbH (HZI), Inhoffenstrasse 7, 38124 Braunschweig, Germany; joachim.wink@helmholtz-hzi.de

**Keywords:** *Actinobacteria*, date palms rhizosphere, saline environments, antimicrobial activity

## Abstract

The diversity of cultural *Actinobacteria* in two types of Algerian Sahara environments, including saline environments and date palms rhizosphere, was investigated. In this study, a total of 40 strains of actinomycetes was isolated from different soil samples, using a rehydration and centrifugation method. Molecular identification, based on 16S rRNA gene sequence analysis, revealed that these isolates were affiliated to six clusters corresponding to eight genera, including *Streptomyces, Nocardiopsis, Saccharopolyspora, Actinomadura, Actinocorallia, Micromonospora*, *Couchioplanes,* and *Planomonospora*. A taxonomic analysis, based on the morphological, physiological, biochemical, and molecular investigation, of selected strains, which belong to the rare *Actinobacteria*, was undertaken. Four strains (CG3, A111, A93, and A79) were found to form distinct phyletic lines and represent new actinobacterial taxa. An assessment of antimicrobial proprieties of the 40 obtained actinomycetes strains, showed moderate to strong antimicrobial activities against fungi and bacteria. This study demonstrated the richness of Algerian Sahara with rare *Actinobacteria*, which can provide novel bioactive metabolites, to solving some of the most challenging problems of the day, such as multi-drug resistance.

## 1. Introduction

With the uncontrollable increase of bacterial resistance to antibiotics [1], the emergence of different types of cancer, as well as the prevailing of viral diseases, such as AIDS and hepatitis C, and other serious diseases [2], the discovery of new drugs become a global emergency.

Actinomycetes are aerobic gram-positive bacteria with high G+C [3]. The microscopic appearance of this group of bacteria can range from coccoid-shaped forms to a thin filamentous branching mycelium with 1–2 µm in diameter [4]. At maturity, the mycelium can produce particular structures likes, spores, arthrospores, conidia, and sporoangia, which are extremely important in the taxonomy of this group of microorganisms [5]. Actinomycetes spores are generally resistant to several physicochemical parameters, such as radiation, desiccation, heating, and a wide range of chemical agents. Such property can be used for selective isolation of some actinomycete taxa [4,5].

The chemical composition of the cell wall and plasma membranes is an essential tool in the classification of *Actinobacteria*. This includes the identity of the amino acid in position three of the tetrapeptide side chain of peptidoglycan, the presence or absence of glycine in interpeptide bridges, the composition of polar lipids and fatty acids of plasma membranes, as well as the composition in the sugar of whole-cell hydrolytes [6].

Actinomycetes are predominantly found in soil, freshwater, and marine environment [7]. Some species, such as *Mycobacterium tuberculosis, Nocardia* spp., and *Actinomyces* spp. are human opportunistic pathogens, and can cause infectious diseases like tuberculosis, *nocardiosis*, as well as actinomycosis, respectively [8,9].

Actinomycetes are the most important group of microorganisms in the field of biotechnology, as producers of various useful secondary metabolites, such as antibiotics [10], antifungal [11], antitumor [12], immunosuppressive, vitamins, and anti-inflammatory agents [13]. One of the main successful potential approach to meet the ever-growing requirement for such compounds, is the exploration of secondary metabolites secreted by rare *Actinobacteria* (non-*Streptomyces Actinobacteria*). However, the isolation of these groups of bacteria is usually difficult, due to their special growth requirements or unknown culture conditions [14]. For this, several selective methods have been developed to facilitate the frequency of isolation of rare *Actinobacteria* from untapped ecological niches, which may lead to the identification of novel strains with a new gene cluster, and hence, new bioactive products [15].

The Algerian Sahara is a part of the largest hot desert in the world, encompassing over 3.5 million square miles, which corresponding to 90% of the Algerian territory. This unique ecosystem is characterized by low rainfall and variable temperature that can change drastically from day to night, in fact, the average daytime temperature is around 38 °C and can get down to −4 °C at night. Furthermore, the Algerian desert is exposed to severe solar radiation during the day [16,17]. Microorganisms, including actinomycetes, which survive under such harsh environmental conditions, acquire the ability to biosynthesize diversified natural bioactive compounds [18].

Studies by the team of Sabaou et al., since 2000, indicating the richness of Algerian Sahara habitats with the novel strains of *Actinobacteria* [19,20,21], and their great potential for the production of bioactive compounds. Boudjella et al., 2010 [22] purified from the strain of *Streptosporangium* sp. Sg3, a new antimicrobial compound that belongs to the class of angucyclinone. Recently, two new derivatives of the antibiotic angucycline, called Mzabimycins A and B, have been obtained from the strain *Streptomyces* sp. PAL114 [23]. In this sense, the aims of this study were to investigate the biodiversity of culturable *Actinobacteria* isolated from two environments, such as saline environments and date palms rhizosphere, located in different regions of the Algerian Sahara, as well as to evaluate their potential to exhibit antimicrobial activities against different target microorganisms.

## 2. Materials and Methods

### 2.1. Sample Collection

Soil samples were collected from two types of environments, including: (i) Saline environments, such as saltpans, saline lake, salt mountain, and (ii) date palms rhizosphere. The sampling areas are located in different regions of Algerian Sahara (Table 1). The samples were taken at a depth of 15–20 cm below the surface, and packed in sterile polyethylene bags, and then stored at 4 °C for further analysis.

### 2.2. Isolation of Actinomycetes

All samples were air-dried, at room temperature, for seven days, to reduce the vegetative bacterial microbiota [17]. After drying, the samples were enriched by the rehydration and centrifugation (RC) method, which led a rapid and selective isolation of diverse zoosporic actinomycete genera directly from the soil [24].

Fifty mL of sterile phosphate buffer (10 mm, pH = 7), containing 10% of soil extract, was gently flooded in a conical beaker (46 mm in diameter and 60 mm deep) containing 0.5 g of air-dried soil. The Erlenmeyer flask was incubated statically at 30 °C for 90 min, to allow for the liberation of motile zoospores. A portion (8 mL) of the suspension was transferred into a screw-cap test tube (16.5 × 105 mm), and centrifuged in a swinging bucket rotor, at room temperature, for 20 min, and at 1.500× *g*. The tubes were allowed to settle on the bench for 30 min at room temperature; afterward, a portion of 1 mL of the suspension containing zoospores was carefully removed from the upper part of the suspension, and serially diluted with sterile tap water. Aliquots of 0.2 mL of each dilution were spread on duplicate onto agar plates of three different medium: Casein Starch Agar (CSA) [17], *Humic acid-vitamin* agar [25], Chitin vitamin B [26]. All media were supplemented with 50 mg/L of cycloheximide to inhibit fungal growth. The plates were incubated at 30 °C for four weeks. The actinomycetes were picked, based on their morphological characteristics, and re-streaked on a new culture medium to obtain a pure culture.

### 2.3. Molecular Identification

#### 2.3.1. DNA Extraction, Amplification, and Sequencing

Molecular identification was carried out based on the sequencing of 16s rDNA. The strains were cultured in GYM medium (glucose 4.0 g/L, yeast extract 4.0 g/L, malt extract 10 g/L, CaCO_3_ 2 g/L, Agar 12 g, Deionized Water 1000 mL) at 30 °C for 3–5 days with constant agitation at 150 rpm. DNA extraction was conducted using, Invisorb Spin Plant Mini Kit (Invitek. GmbH, Berlin, Germany), following the manufacturer’s protocol. The 16S rRNA gene region was amplified by PCR, using two universal primers; forward primers bind on the position 27F [5′-AGAGTTTGATC(AC)TGGCTCAG-3′] and reverse primers bind on the position R1492 [5′-ACGG(CT)TACCTTGTTACGACTT-3′] [27].

The PCR reaction was carried out in microtubes of 200 µL, the total reaction volume is 50 µL: 25.0 μL “JumpStart Ready Mix, SigmaAldrich, Germany” (1.25 units Taq DNA polymerase, 10 mM Tris-HCl, 50 mM KCl, 1.5 mM MgCl_2_, 0.2 mM dNTP, pH = 9, 0.1% Triton X-100, 0.2 mg mL^−1^ bovine serum albumin), 1 μL of each primer (27F and R1492), 22 μL PCR water, and 1 μL genomic DNA. One tube remains without DNA, and therefore, serves as a negative control.

The PCR reaction was conducted in a Mastercycler Gradient (Eppendorf, Hambourg, Germany) using the following program: Initial denaturation at 95 °C for 5 min, 35 cycles of denaturation at 94 °C for 30 s, annealing at 52 °C for 30 s, elongation at 72 °C for 120 s, and a final extension at 72 °C for 10 min. PCR products were purified using NucleoSpin^®^ Gel and PCR Clean-up-Kit (MachereyNagel, Düren, Germany) and eluted in 30 μL of elution buffer. PCR products were checked on an agarose gel (0.8%), the separation was carried out by electrophoresis at 70 V for 50 min in TAE buffer. DNA bands were visualized under UV light, after staining with an ethidium bromide solution.

16S rRNA genes of actinomycetes strains were sequenced using primer 27F and R518. The obtained sequences were checked for quality and assembled using the program ‘‘Bioedit alignment, v 7.0.5.3”. Additional primer (F1100, R1100, R1525) was used to obtain the full 16s rDNA sequence.

The obtained sequences were compared to sequences of the public database (NCBI) using BLAST search (https://blast.ncbi.nlm.nih.gov/Blast.cgi), and EZ-taxon (https://www.ezbiocloud.net/) [28].

#### 2.3.2. 16S rRNA Gene Phylogenetic Analyses

The almost full-length 16S rRNA gene of selected strains was aligned with multiple sequences of the closely related type species retrieved from the GenBank database, using ClustalW programme (versio 2.1, Thompsonet et al, Heidelberg, Germany).

Phylogenetic trees were generated with the neighbor-joining algorithms [29], using molecular evolutionary genetics analysis (Mega) software (version 7.0, Tamura et al, Tokyo, Japan) [30]. The stability of the topology of the phylogenetic trees was evaluated using the bootstrap resampling method with 1000 repetitions [31].

### 2.4. Taxonomic Study of Selected Isolates

Selected strains were subjected to morphological, physiological, biochemical characterization, and compared to the closest types species described according to the compendium of *Actinobacteria*: https://www.dsmz.de/collection/catalogue/microorganisms/special-groups-of-organisms/compendium-of-actinobacteria.

Cultural characteristics were determined after three weeks of incubation at 30 °C, according to the methods described by Shirling and Gottlieb (1966) [32]. The colors of both substrate and aerial mycelia were determined according to the RAL color code (Deutsches Institut für Gütesicherung und Kennzeichnung e.V.—Reichsausschuß für Lieferbedingungen). The spore morphology of selected strains was observed by light microscopy and a scanning electron microscopy, after two weeks of growth at 30 °C on suitable media.

Temperature, pH, and NaCl tolerance were determined in ISP2 as a basal medium, and after two weeks of incubation. NaCl tolerances were determined at different concentrations (0–7.5%, *w*/*v*), whereas, the temperature range for growth was determined at 20, 25, 30, 37, and 45 °C. The pH range for growth was determined at a pH between 4.0–12.0 with an interval of 1.0 pH unit, at 30 °C.

The ability of the selected strains to use ten different carbon sources (glucose, arabinose, saccharose, xylose, inositol, mannose, fructose, rhamnose, raffinose, cellulose) was conducted at 30 °C, as described by Shirling and Gottlieb, 1966 [32]. The final concentration of each sugar was adjusted to 1%, one plate containing only pure basal medium and water is used as a negative control, while the one with the glucose serves as a positive control. Enzymatic activities, including nitrate reductase, ureas, gelatin liquefaction, H_2_S production, were determined using API^®^ CORYNE, API^®^ CAMPY, and API^®^ Zym test strips (bioMerieux, Marcy-l’Étoile, France).

### 2.5. Evaluation of Antimicrobial Activity

#### 2.5.1. Preparation of Suspension

For preliminary screening of antimicrobial activity, seven bacteria, such as: *Bacillus subtilis* DSM10, *Staphylococcus aureus* Newman, *Micorococcus luteus* DSM1790, *Pseudomonas aeruginosa* PA14, *Escherichia coli* TolC, *Klebsiella pneumoniae ATCC,* and one yeast *Candida albicans* DSM1665, were used as test microorganisms. However, for secondary screening, in addition to the test microorganisms used in preliminary screening, two more bacteria (*Mycobacterium smegmatis* ATCC700084 and *Chromobacterium violaceum* DSM30191) and two fungi (*Mucor himalis* DSM2656, *Pichia anomala* DSM6766) were tested.

For the preparation of suspension for antimicrobial activity, bacterial and fungi test microorganisms were inoculated in Mueller Hinton Broth (MHB), and Sabouraud Dextrose Broth (SDB), respectively. After 24 h incubation at 37 °C for bacteria, and 48 h incubation at 30 °C for fungi, the optical density for both suspensions were adjusted between 0.08–0.1 (measured at 600 nm), which corresponded to the microbial density between 10^6^ CFU/mL–10^8^ CFU/mL.

#### 2.5.2. Preliminary Screening for Antimicrobial Activity

The actinomycetes strains were inoculated on ISP2 plates medium (glucose 4.0 g/L, yeast extract 4.0 g/L, malt extract 10 g/L, Agar 12 g, deionized water 1000 mL), the plates were then incubated at 30 °C for 14 days. Cylinders pieces (6 mm in diameter) were cut from the well-grown sporulated culture of actinomycetes strains, and then placed on the surface of Muller Hinton plats already seeded with the test microorganisms. The plates were kept at a temperature of 4 °C for 4 h, to ensure a good diffusion of secondary metabolites secreted by actinomycetes strains. Afterward, the plates were incubated at an appropriate temperature. The zones of inhibition were determined after 24 h (bacteria and yeasts) and 48 h (fungus) with the Haloes Caliper (IUL Instruments, Barcelona, Espagne) [33].

#### 2.5.3. Secondary Screening for Antimicrobial Activity

The mature spores of actinomycetes strains, grown in GYM medium, were inoculated in Erlenmeyer flasks (250 mL) which contained 100 mL of 5294 medium (10 g/L starch, yeast extract 2 g/L, glucose 10 g/L, glycerol 10 g/L, corn steep liquor 2.5 g/L, peptone 2 g/L, Nacl 1g/L, CaCO_3_ 3 g/L, distilled water 1 mL, pH = 7.2). The flasks were incubated at 30 °C and 160–180 rpm, for two weeks, in a rotary shaker. Twenty mL of this culture was transferred in 50 mL of Falcon tubes, which contained 20 mL of ethyl acetate. The tubes were mixed for 20 min on a rotary shaker. Afterward, the tubes were centrifuged at 9000 rpm for 10 min, and the upper phase is transferred into a 50 mL round bottom flask. The ethyl acetate is evaporated at 40 °C, in a rotary evaporator, and the organic extract is solved in 1 mL methanol and centrifuged at 14,000 rpm for 10 min [34].

Antimicrobial activity was determined by serial dilution method, in 96-well plates, with EBS medium (0.5% casein peptone, 0.5% glucose, 0.1% meat extract, 0.1% yeast extract, 50 mM HEPES [11.9 g/L] and pH 7.0) for bacteria and MYG medium (1.0% phytone peptone, 1.0% glucose, 50 mM HEPES [11.9 g/L] pH 7.0) for fungi. Oxytetracycline and nystatin were used as a positive control for antibacterial and antifungal activity, respectively, while methanol was used as a negative control.

## 3. Results and Discussion

### 3.1. Actinomycetes Isolation

Forty actinomycete strains were isolated from soil samples collected from two Algerian Sahara environment, such as date palms rhizosphere and saline environments, using, centrifugation-rehydration method.

According to molecular identification results, the 40 strains of actinomycetes were affiliated to 6 clusters, belong to nine different genera, including: *Actinocorallia* (1), *Actinomadura* (1), *Micromonospora* (2), *Couchioplanes* (1). *Planomonospora* (1), *Saccharopolyspora* (1), *Nocardiopsis* (10), and *Streptomyces* (23). The results are shown in Table 2.

#### 3.1.1. Cluster I

This cluster is the largest group; include 23 isolates of actinomycetes, which belong all to the genus of *Streptomyces*. This result is consistent with previous studies, which reported that *Streptomyces* was the most predominant genus of *Actinobacteria* in the soil [35,36].

Ten isolates, C119, V11, V9, A34, A1, O2, A36, A16, A10, A4, were assigned, with 100% similarity, to the type strains belong to the genus *Streptomyces*. While the percentage of similarity for the other 13 isolates affiliated to the genus *Streptomyces*, range from 99.25%, for the isolate CAC512 with *Streptomyces qinglanensis*, to 99.93% for the strain C56 with *Streptomyces olivaceus*.

All strains belong to this cluster form branched substrate mycelium, which is rarely fragmented. At maturity, the aerial mycelium forms chains of 3–20 spores [37].

Members of the genus *Streptomyces* represent the first source of secondary metabolites from the microbial origin [38]. Indeed, according to the database ‘dictionary of natural products’ (CRC press; Taylor and Francis group), 7953 molecules have been isolated from this genus. For this, the probability of obtaining new compounds from the genus *Streptomyces* has become increasingly low, due to the mechanism of genetic exchange between the strains in the environment [39]. Consequently, the actual trend is oriented towards exploiting secondary metabolisms of rare *Actinobacteria* [40].

#### 3.1.2. Cluster II

This cluster is represented by ten isolates, which belong all to the genus of *Nocardiopsis* (Table 2). All strains of this cluster were isolated from a different saline environments such as: Saltpan of Hassi Bah bah (Djelfa), the saline lake of Ain Ouarka (Naama), saltpan of Bougtob (El-Bayadh), saltpan of Kenadsa (Bechar), and salt Mountain of Kaf el melh (Laghouat). Indeed, the species belongs to the genus *Nocardiopsis* are known for their tolerances to high NaCl concentrations, and they are abundant in the saline areas [41].

The isolates belongs to this cluster form a dense and branched, well-developed substrate mycelium which fragments, at maturity, into rod-shaped and non-motile spores; however, the aerial mycelium breaks up into chains of straight, branched, or zigzag spores. This microscopic morphology typically characterizes the genus *Nocardiopsis* [42,43].

According to the molecular identification, five isolates (T1, BO15, C10, C19, and CHB2), are close to the species *Nocardiopsis terrae* with different similarity values (Table 1), while the strains, T14 and A58, has a similarity of 100% with the species, *Nocardiopsis halotolerans* and *Nocardiopsis arvandica*, respectively.

**Figure 1:**
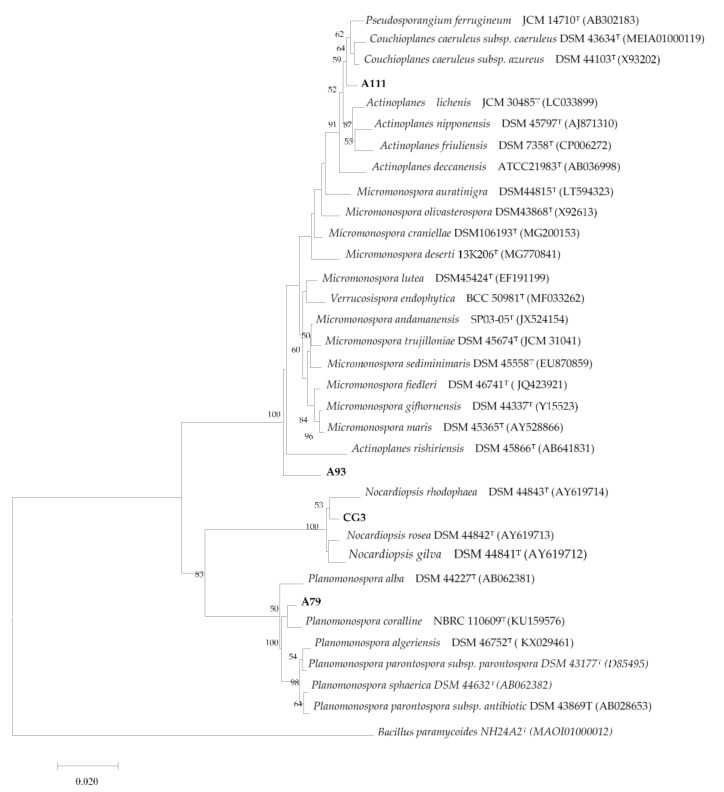
Neighbor joining phylogenetic tree based on almost-complete 16S rRNA gene sequences showing the position and phylogenetic relationship between the strains, A93, CG3, A111, A79, and the type strains of the closest species. Numbers at the nodes are bootstrap values; expressed as a percentage of 1000 resamplings (only values 50% are shown).

The strain CG3 show low similarity (99.20%) with the species *Nocardiopsis rosea.* The phylogenetic tree, Figure 1, revealed that the isolate CG3 with the species, *Nocardiopsis gilva* YIM 90087, *Nocardiopsis rosea YIM* 90,094, and *Nocardiopsis rhodophaea* YIM 90096, form a distinct clade within the tree. These results were supported by the high bootstrap values and should be considered significant.

**Figure 2:**
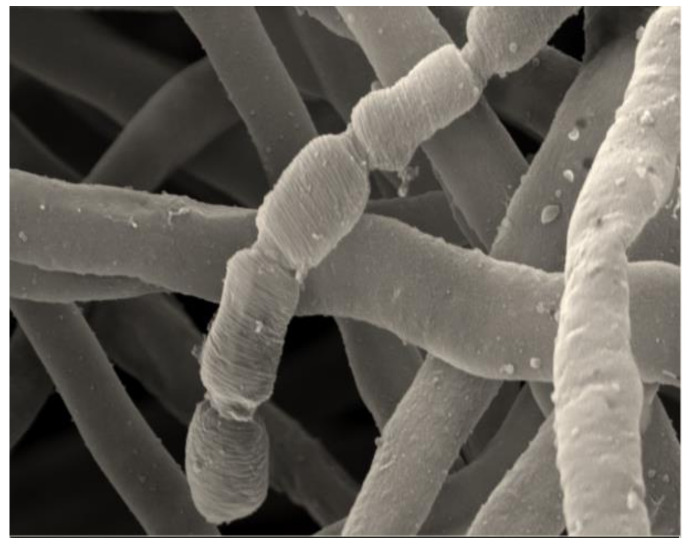
Micromorphology of aerial mycelium, seen by scanning electron microscopy, of the strain CG3, showing short and straight spore chains with a smooth surface, after growth on ISP3 medium supplemented with 3% of Nacl and incubated for 18 days at 37 °C. scale: 200 nm.

The strain CG3 was isolated from the soil of Kenadsa’s saltpan (Bechar region, south-west Algeria), using Starch Casein Agar (SCA) medium. The aerial mycelium, is well developed and carries short spore chains with a smooth surface (Figure 2). The substrate mycelium is stable, unlike the other species of the genus *Nocardiopsis* that form fragmented substrate mycelium.

Morphological, physiological, and biochemical properties of strains CG3 is shown in Table 3.

The aerial mycelium of the isolate CG3 is white on all ISP used, while the substrate mycelium varied from golden yellow-zinc yellow. However, the closest species to the strain CG3, *Nocardiopsis rosea*, form a pink white-pale pink aerial mycelium, while the substrate mycelium is pale pink-moderate red on media tested. No diffusible or melanoide pigments were produced on all ISP media used for the strain CG3, as well as *Nocardiopsis rosea* [44].

Several biochemical differences were noted between the strain CG3 and the closest species *Nocardiopsis rosea,* in fact, among the 10 carbon sources tested; the species *Nocardiopsis rosea* can use lactose, rhamnose, arabinose, and cellulose [44], unlike the isolate CG3, which cannot use them. However, the strain CG3 can use, inositol, but the species *Nocardiopsis rosea* cannot use it. Both have a similar metabolism toward the other carbon sources (glucose, saccharose, xylose, mannose, fructose, and raffinose). Other biochemical differences have been reported, the strain CG3 is positive for lipase (C14), chymotrypsine, cystine arylamidase, trypsine, β-glucosidase, and urease, as well as can liquefy gelatin, unlike the species *Nocardiopsis rosea,* which is negative for all these tests [44].

For physiological tests, the temperature growth of the strain CG3 range between 25–50 °C (with an optimum at 37 °C), however the species, *Nocardiopsis rosea,* can grow at a temperature range between 20–60 °C (with an optimum at 37 °C). Both have the same tolerance toward NaCl (0–18)%, with the same optimum concentration of growth: (3–7)% NaCl. For pH, the isolate CG3 growth between 6–12, while the species *Nocardiopsis rosea* growth between 6–9, and both have the same optimum pH growth (pH = 7).

Basis to the results of molecular identification and phylogenetic study, as well the differences observed in biochemical, physiological, and morphological characteristics, the strain CG3 can represent a novel species within the genus *Nocardiopsis*, under a reserve of DNA-DNA hybridization. Indeed, the percentage of similarity between the strain CG3 and the closest species, *Nocardiopsis rosea*, is superior to the cutoff value (98.6%) for differentiating two bacterial species, as reported by [28].

#### 3.1.3. Cluster III

Molecular identification indicates that the only members of cluster III, strain S27, is 100% similar to the species *Saccharopolyspora erythraea*.

The strain S27 was isolated from the sediments of the salt lake of Ain-ourka (wilaya of Naama, west of Algeria), using chitin vitamin B medium. Microscopic observation of the strain S27 indicates that this isolate form a branched substrate mycelium, which fragments, at maturity, into coccoid and bacillary elements, the aerial mycelium of this strain forming a spore chain surrounded by a sheath. This micromorphology is typical for the strains that belong to the species of *Saccharopolyspora erythraea* [45]. According to these results, the isolate S27 should be identical to the species *Saccharopolyspora erythraea.*

#### 3.1.4. Cluster IV

This cluster is represented by three isolates, A15 A111 A93, which belong all to the family of *Micromonosporaceae*. These strains were isolated from the Ain-Salah’s palms rhizosphere, using humic acid-vitamin B medium, after enrichment of the soil samples by the rehydration-centrifugation method.

Molecular identification indicates that the strain A93 was close to the species *Micromonospora yasonensis*, with 99.38% similarity, while the isolate A111 was close to the species *Couchioplanes caeruleus subsp. azureus,* with 98.74% similarity. Phylogenetic tree, Figure 1, indicate that the strain A111, form a distinct branch within the cluster formed by the species *Pseudosporangium ferrugineum*, *Couchioplanes caeruleus subsp. azureus*, and *Couchioplanes caeruleus subsp. Caeruleus*.

**Figure 3:**
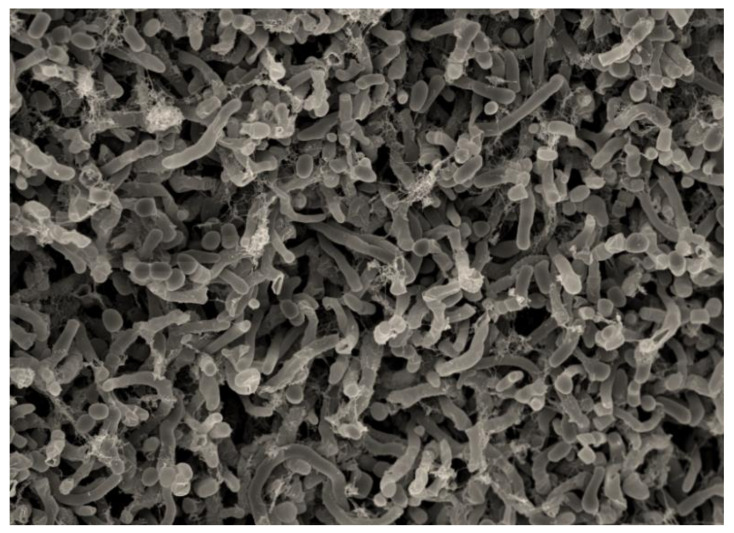
Scanning electron microscopic observation of the strain A111, showing short chains of arthrospores (3–5) with smooth surfaces, after growth for 14 days at 30 °C on ISP2 medium. Scale: 200 nm.

Aerial mycelium is absent for the strain A111, however, scanning electron microscopic observation of the substrate mycelium of this strain, showed the formation of short chains of motile arthrospores (3–5) with smooth surfaces (Figure 3), while sporangia are not observed. This description corroborates perfectly with the microscopic morphology of the genus *Couchioplanes* [39]. This is the first report of the isolation of strain belongs to the genus *Couchioplanes*, from Algerian soil, in fact, this genus only includes one species—*Couchioplanes caeruleus*.

Several differences were noted between the isolate A111 and the closest species *Couchioplanes caeruleus subsp. azureus*. The substrate mycelium of the strain A111 is orange on ISP (2, 3, and 4), colorless on ISP5, honey-yellow on ISP6, and pearlescent copper on ISP7, unlike the species *Couchioplanes caeruleus subsp. azureus*, which produces a dark green-dark blue substrate mycelium. The strain A111 form honey-yellow soluble pigments on ISP6 medium and pearlescent copper soluble pigments on ISP7 medium, whereas, the species *Couchioplanes caeruleus subsp. azureus* does not produce any soluble pigment [46].

In addition to the morphological differences, biochemical differences have been observed, in fact, the isolate A111, can use the ten tested sugars as the only carbon source, whereas the species *Couchioplanes caeruleus subsp. azureu,* uses only glucose, fructose, inositol, and sucrose. Nitrate reductase and gelatin liquefaction are positive for both [46]. However, the strain A111, is positive for alkaline phosphatase, esterase (C4), esterase lipase (C8), lipase (C14), leucine arylamidase, valine arylamidase, cystine arylamidase, trypsin, a-chymotrypsin, acid phosphatase, naphthol-ASBI-phosphohydrolase, α-galactosidase, β-galactosidase, α-glucosidase, β-glucosidase, N-acetyl-b-glucosaminidase, and β-glucuronidase.

For physiological tests, both have the same tolerance toward NaCl 0%–2.5%, the same optimal temperature growth (30 °C), as well as both, have the same optimum pH growth (pH = 7).

By considering the results of molecular identification and phylogenetic study, in addition to the differences observed in morphological and biochemical characteristics, between the strain A111 and the closest species, *Couchioplanes caeruleus subsp. azureus*, we can conclude that the strain A111, can be a new member within the genus *Couchioplanes*, under a reserve of DNA-DNA hybridization.

Molecular identification indicates that the strain A93 was close to the species *Micromonospora maris*, with 97.56% similarity, whereas, the phylogenetic analysis, Figure 1, inferred from 16S rRNA gene, indicated that this isolate formed a distinct phylogenetic line within the phylogenetic tree.

**Figure 4:**
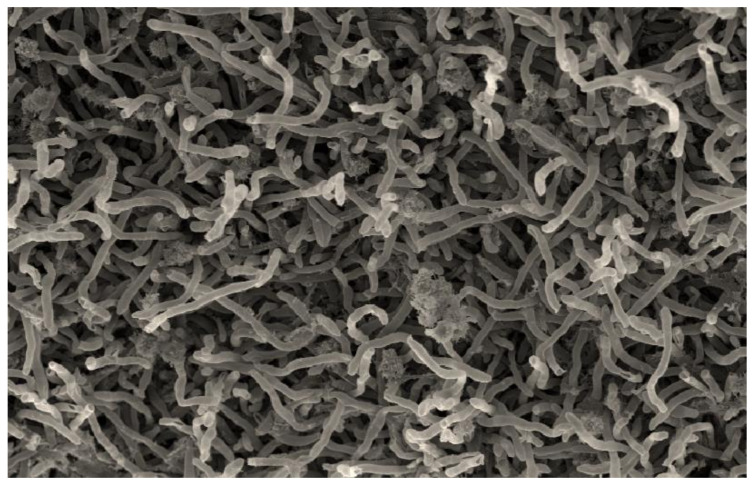
Scanning electron microscopy observation of the strain A93 showing short chains of arthrospores surrounded by a sheath, after growth for 14 days at 30 °C on ISP2 medium. Scale: 200 nm.

The typical microscopic morphology of the genus *Micromonospora* is characterized by the formation of single and non-motile spores in the substrate mycelium [46]. However, the microscopic morphology of the strain A93 (Figure 4), observed by scanning electron microscopy, is different compared to the genus *Micromonospora*. In fact, the single spores have not been observed in the substrate mycelium, whereas, short spore chains surrounded by a smooth sheath are formed in the substrate mycelium of the isolate A93 (Figure 4).

The isolate A93 can grow only on ISP2, ISP3, ISP6, and ISP7, without forming an aerial mycelium, whereas, the substrate mycelium is either narcissus yellow on ISP2 or colorless on ISP3, ISP6, and ISP7, however, the strain A93 cannot grow on ISP4 and ISP5.

For biochemical characteristics, the strain A93 can use, glucose, xylose, inositol, mannose, fructose, rhamnose, and raffinose as sole carbon source, whereas, cellulose, arabinose, and sucrose cannot be used. Nitrate reductase is positive, while liquefaction of gelatin and urease are negative for this strain.

The strain A93 is positive for alkaline phosphatase, esterase (C4), esterase lipase (C8), lipase (C14), leucine arylamidase, valine arylamidase, cystine arylamidase, trypsin, a-chymotrypsin, acid phosphatase, naphthol-ASBI-phosphohydrolase, α-galactosidase, β-galactosidase, α-glucosidase, β-glucosidase, N-acetyl-b-glucosaminidase, α-mannosidase, α-fucosidase, whereas is negative for β-glucuronidase.

The strain A93 growth at a temperature range from 25–35 °C, and at a pH range between 6–9, as well as in the presence of 0%–2.5% (*w*/*v*) NaCl. The optimal growth is observed at 30 °C, pH = 7 and 0% (*w*/*v*) NaCl.

Based on the low percentage of similarity (97.56%), between the isolate A93 and the closest species, *Micromonospora maris*. Moreover, the phylogenetic tree, which showed that the strain A93 form a distinguishable, monophyletic, and stable branch (bootstrap value > 80%) to the branches comprising the other genera belong to the family *Micromonosporaceae*. Therefore, these results suggest that the strain A93 does not belong to *Micronomospora*, and can be affiliated to a new genus within the family of *Micromonosporaceae.* This finding should be confirmed by further detailed chemotaxonomic analysis.

#### 3.1.5. Cluster X

One isolate, A79, belong to this cluster. This strain was isolated from the palms of Ain-Salah, using the humic acid-vitamin B medium, after enrichment of the soil by the rehydration-centrifugation method. Molecular identification indicates that the isolate A79 is close to the species *Planomonospora corallina* with 98.90% similarity.

Phylogenetic studies pointed out that the isolate A79 clustered clearly with the species, *Planomonospora coralline*.

**Figure 5:**
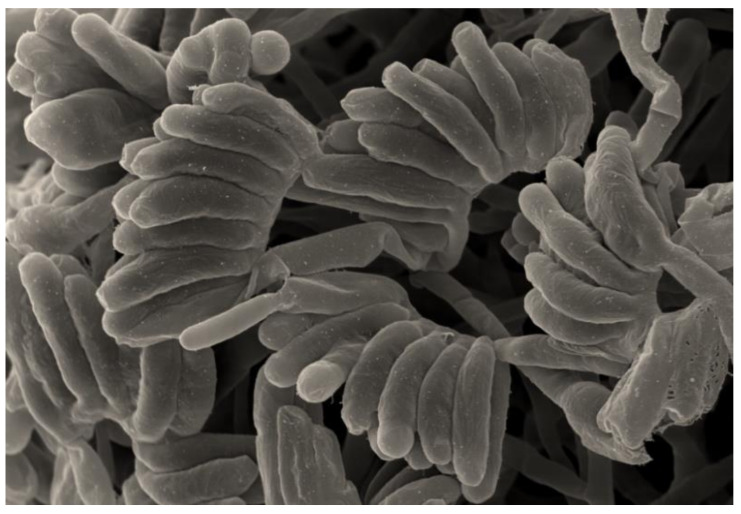
Scanning electron microscopy observation of the strain A79, showing sporangia arranged in two parallel rows resembling rows of bananas, after growth for 14 days at 30 °C on the medium ISP3. Scale: 200 nm.

The strain A79 showed characteristics typical of the genus *Planomonospora*. It was gram-staining-positive and non-acid-fast. Scanning electron microscopy observation, showed that the aerial hyphae of the strain, A79, produced sporangiophores bearing long parallel rows of large sporangia which resembled rows of bananas (Figure 5); therefore, it belongs to the group of *P. parontospora* [47]. The spores formed by the isolate A79 are motile, as observed with a light microscope, after incubating the sporangia in 0.1 M potassium phosphate buffer (pH = 8) at 30 °C for 30–60 min. Fragmentation of substrate mycelium was not observed.

The comparison of the cultural characteristics of the strain A79 with the closest species, *Planomonospora corallina,* showed the existence of several macromorphological differences, in fact, the isolate A79 can grow only on ISP3, ISP4, and ISP7. The aerial mycelium of this strain was cream on ISP3, colorless on ISP7, and absent on ISP4, unlike the species *Planomonospora corallina*, which has good growth on both media, ISP2 and ISP5 [48]. The substrate mycelium were light orange-yellowish pink for the species *Planomonospora corallina*, however, it was creme for the isolate A79. The soluble pigment was not observed for *Planomonospora coralline*, as well as the strain A79.

The differences between the strain A79 and the species *Planomonospora corallina* extend to the biochemical characters. The species *Planomonospora corallina* can use glucose, fructose, rhamnose, xylose, cellulose, but cannot use arabinose, inositol, mannose, or sucrose, [48], unlike the isolate A79, which can use glucose, arabinose, sucrose, xylose, inositol, mannose and fructose, but not raffinose, cellulose, and rhamnose. The isolate A79 is positive for the test of naphthol-AS-BI-phosphohydrolase, but the species *Planomonospora coralline* is negative for this test.

The strain A79 growth at a temperature varied between 25–35 °C (optimum 30 °C), tolerates NaCl concentrations between 0%–2.5% NaCl (*w*/*v*), and growth at pH range between 6–9 (optimum pH: 7–8). While the closest species, *Planomonospora coralline,* growth at a temperature range between 20–37 °C (optimum 25–30 °C), pH 6–11 (optimum pH: 8–9), however, this species could not sustain growth with 1% NaCl [48].

According to the obtained results, we can conclude that the strain A79 represent a new species within the genus *Planomonospora*, under a reserve of DNA-DNA hybridization.

#### 3.1.6. Cluster IX

This cluster is represented by two isolates, A23 and A112. Sequencing of 16S rDNA indicates that the isolate A23 was close to *Actinocorallia libanotica* with 99.86%, while the similarity between the isolate A112 and the closest species, *Actinomadura montaniterrae,* was 99.33%. Both genera, *Actinocorallia* and *Actinomadua*, are a member of the family *Thermomonosporaceae*.

The strains, A23 and A112, were isolated from Ain-Salah’s palms, using humic acid-vitamin B medium, after enrichment of soil by a rehydration-centrifugation method.

The strains, A23 and A112, showed macroscopic and microscopic characteristics typical of the species belongs to the genus *Actinocorallia* and *Actinomadua*, respectively. The isolate A23, forms pale yellowish, well developed branched, non-fragmenting vegetative mycelium. Mature aerial mycelium is pink, and carries short ellipsoidal spore chains. However, the strain, A112, forms an extensively branched and non-fragmented substrate mycelium. The aerial mycelium is cream, and forms short straight chains of arthrospores.

The sequences of 16S rRNA gene of the strains, A111, A79, A93, and CG3, obtained in this study, were deposited in GenBank under the accession numbers: MT259018; MT258992, MT259019, and MG972881, respectively.

### 3.2. Antimicrobial Activity of Isolated Strains

#### 3.2.1. Preliminary Screening

Sixteen isolates, among 40 tested strains, showed antimicrobial activity against at least one of the target microorganisms (Table 4). Among them, twelve strains were active against at least one of the gram-positive bacteria, and six isolates were active against at least one of the gram-negative bacteria; in addition, five strains exhibited antibacterial activity against both, gram-positive and gram-negative bacteria, however, six strains showed antifungal activity against the yeast *Candida albican.*

The results indicate clearly that gram-positive bacteria were more sensitive to secondary metabolites secreted by the actives strains of *Actinobacteria*, when compared to gram-negative bacteria. This dissimilarity could be ascribed to the morphological differences between these two groups of bacteria, in fact, the outer membrane of gram-negative bacteria having lipopolysaccharide, which makes the cell wall impermeable to the lipophilic extracts. However, the gram-positive bacteria were more susceptible, due to the lack of outer membrane [49].

Eight actives strains, such as C56, RH94, S29, C7, V12, V9, V5, and A28, belong to the genus *Streptomyces*, among them, the isolate V12 was the most active strain and shows activity against all target microorganisms. These results are in correlation with the previous studies, which indicated that the genus *Streptomyces* is the major source of bioactive metabolites with antimicrobial activity [38,50].

Three actives isolate (CG3, BO15, and T1) were affiliated to the genus *Nocardiopsis*. Several studies reported that this genus is a potential source of *bioactive compounds*, which display a wide spectrum of pharmacological and biological effects, including antibacterial [51], antifungal [52], anticancer [53], and immunomodulatory [54]. The isolate, CG3, show a broad spectrum of antimicrobial activity against all target microorganisms tested, except against *Klebsiella pneumoniae*. Recently, five new compounds, kenalactams A-E, belong to the class of polyenic macrolactams that have been purified from the crude extract of the strain CG3. These new compounds showed cytotoxic activity against a panel of five human cancer cell lines, including HeLa cells KB3.1, human lung carcinoma A549, ovarian carcinoma SKOV-3, human prostate cancer PC-3, human breast adenocarcinoma MCF-7, and normal cell line mouse fibroblasts L929 [55].

#### 3.2.2. Secondary Screening

Based on the results of preliminary screening, as well as the results of molecular identification, six strains (S27, A23, CG3, A15, A111, A79), among 40 isolates, were selected for secondary screening of antimicrobial activity. The results are shown in Table 5.

The selected strains, S27, A23, CG3, A15, A111, A79, belong to the rare genus of *Actinobacteria*, including *Saccharopolyspora, Actinocorallia, Nocardiopsis, Micromonospora, Couchioplanes*, and *Planomonospora*, respectively. These strains exhibit antimicrobial activity against, at least, one of the target pathogenic microorganisms used in the preliminary screening.

The isolate, CG3, showed the strongest antimicrobial activity against the eight-target microorganisms used, with MIC values varied between 0.52–66.66 μg/mL. However, the strains A79 and 111, showed moderate to weak antimicrobial activity. In fact, the isolate A111, was active against the two bacteria, *Staphylococcus aureus*, and *Chromobacterium violaceum,* with MIC values of 8.33 µg/mL and 66.66 µg/mL, respectively, while the isolate A79 was active against three bacteria, including two gram-negative (*Chromobacterium violaceum* and *Escherichia coli TolC,* with the same MIC value: 33.33 µg/mL) and one gram-positive bacteria (*Staphylococcus aureus,* MIC: 66.66 µg/mL), as well as one fungi (*Mucor hiemalis*, MIC: 66.66 µg/mL). Moreover, the strain A23 exhibited interesting antimicrobial activity against the two gram-positive bacteria, *Staphylococcus aureus*, and *Micrococcus luteus*, with the same MIC value (4.16 µg/mL), as well as shown moderate activity against the gram-negative bacteria, *Escherichia coli* TolC with MIC = 33.33 µg/mL. Furthermore, the two *Actinobacteria* strains, S27 and A15, display antimicrobial activity against *Staphylococcus aureus* (MIC = 66.66 µg/mL) and *Candida albicans* (MIC = 66.66 µg/mL), respectively.

The six selected strains belong to the rare *Actinobacteria*, which are widely reported in the literature as an unexplored source of new bioactive secondary metabolites [56]. The strain CG3 belong to the genus *Nocardiopsis*, members of this genus are known for the production of a variety of bioactive secondary metabolites [52]. The strains A79, A111, and A23, displayed weak to moderate antimicrobial activity, and they belong to the rare genus of actinomycetes, such as *Planomonospora*, *Couchioplanes*, and *Actinocorallia*, respectively. Those genera are rarely mentioned in the literature as a producer of secondary active metabolites, in fact, only one compound, planosporicin has been isolated from the genus *Planomonospora*, however, from the genus *Couchioplanes,* one compound, antibiotic 67-121D, has been purified [57,58]. Moreover, three new compounds, aurantiadioic acids A-B and aurantoic acid A, were obtained from the rare actinomycete, *Actinocorallia aurantiaca* [59].

Several studies indicate that the bioactive compounds secreted by the rare *Actinobacteria*, appear to be encoded by silent or poorly expressed genes under standard cultivation conditions [60]. In fact, the genome of rare *Actinobacteria* enclosed several cryptic biosynthetic gene clusters, which represent a potential source of new scaffolds for the discovery of novel bioactive compounds [61].

This finding has been confirmed through genome sequencing of rare *Actinobacteria*, which revealed a difference between their potential and the expression of their biosynthetic genes [61]. Diverse methods have been developed for the activation of silent or poorly expressed cryptic gene clusters. Among these methods, the changing of the environment in which the microorganism is growing to activate the expression of the silent biosynthetic gene clusters [62]. This can be achieved simply by changing variables, such as temperature and pH [63], or by adding competing species (co-cultivation method) [64], or by the use of chemical elicitors compounds [65].

Among the eight test bacteria, *K. pneumoniae,* showed resistance towards the crud extracts of the five selected actinomycetes strains, whereas, only one isolate, CG3, was found to possess weak antimicrobial activity against *P. aeruginosa* (MIC: 66.66 µg/mL). Previous studies reported that *K. pneumoniae* and *P. aeruginosa*, have a remarkable ability to exhibit natural or acquired resistance to antimicrobial compounds [66,67]. The acquired resistance is may be due to the mutational event or to the acquisition of resistance gene via horizontal gene transfer [68].

## 4. Conclusions

Rare *Actinobacteria* become a new trend and attractive approach for the discovery of useful and novel bioactive secondary metabolites with medical applications. In this study, we investigate the diversity and antimicrobial activity of rare *Actinobacteria* isolated from two types of environment, such as palms rhizosphere and saline environments, located in the Sahara of Algeria. A total of 40 *actinobacterial* strains was identified and affiliated to nine genera. Four strains (CG3, A79, A111, and A93) were considered as new *Actinobacteria* taxa. Sixteen isolates display antagonistic activities against, at least, one of the tested microorganisms; however, the crude extract of the five selected *Actinobacteria* strains (CG3, A79, A111, A15, S27, and A23) exhibited different inhibitory activities against tested microorganisms. This study demonstrated that *Actinobacteria* are adapted to diverse ecological habitats, such as saline environments and palm rhizosphere. In addition, the unexplored ecosystems are a rich source of new taxa of rare *Actinobacteria*, which can provide novel metabolites that may help in solving some of the most challenging problems of the day, such as *multi*-*drug resistance.*

## Figures and Tables

**Table 1:** Location of soil samples collection sites.

Sampling Site	Location	Type of Environnement
Hassi Bah bah (Djelfa)	34°59′16.598″ N 2°58′26.598″ E	Saltpan
Ain Ouarka (Naama)	32°56′21.415″ N 0°3′39.874″ W	Saline lake
Bougtob (El-Bayadh)	34°4′2.377″ N 0°6′45.688″ E	Saltpan
Kenadsa (Bechar)	31°30′40.693″ N 2°14′44.427″ W	Saltpan
kaf el melh (Laghouat)	33°38′47.120″ N 1°51′6.720″ E	Salt Mountain
Ain Salah (Tamanrasset)	27°12′9.533″ N 2°29′16.764″ E	Date palms rhizosphere

**Table 2:** Molecular identification of actinomycete isolates.

Strains	Cluster	Closest Types Species	Similarity (%)
C56	Cluster I	*Streptomyces olivaceus*	99.93
C119		*Streptomyces coelicoflavus*	100.00
V17		*Streptomyces spinoverrucosus*	99.72
RH94		*Streptomyces luridiscabiei*	99.86
CS44		*Streptomyces hydrogenans*	99.46
S29		*Streptomyces artemisiae*	99.65
C7		*Streptomyces peucetius*	99.72
V11		*Streptomyces albogriseolus*	100
V12		*Streptomyces lavendofoliae*	99.88
V9		*Streptomyces pactum*	100
V5		*Streptomyces hydrogenans*	99.49
V3		*Streptomyces qinglanensis*	99.67
CAC512		*Streptomyces qinglanensis*	99.25
A34		*Streptomyces afghaniensis*	100
A28		*Streptomyces levis*	99.68
A5		*Streptomyces viridochromogenes*	99.68
A1		*Streptomyces flavotricini*	100
A108		*Streptomyces levis*	99.66
O2		*Streptomyces griseoincarnatus*	100
A36		*Streptomyces erythrogriseus*	100
A16		*Streptomyces coeruleofuscus*	100
A10		*Streptomyces globosus*	100
A4		*Streptomyces coelicoflavus*	100
C10	Cluster II	*Nocardiopsis terrae*	99.65
CG3		*Nocardiopsis rosea*	99.20
T1		*Nocardiopsis terrae*	99.72
C19		*Nocardiopsis terrae*	99.65
M23		*Nocardiopsis dassonvillei subsp. dassonvillei*	99.74
CHB2		*Nocardiopsis terrae*	99.46
T14		*Nocardiopsis halotolerans*	100
S4		*Nocardiopsis synnemataformans*	99.89
BO15		*Nocardiopsis terrae*	99.72
A58		*Nocardiopsis arvandica*	100
S27	Cluster III	*Saccharopolyspora erythraea*	100.00
A23	Cluster IV	*Actinocorallia libanotica*	99.86
A112		*Actinomadura montaniterrae*	99.33
A15	Cluster V	*Micromonospora yasonensis*	99.38
A111		*Couchioplanes caeruleus subsp. azureus*	98.74
A93		*Micromonospora maris*	97.56
A79	Cluster VI	*Planomonospora corallina*	98.90

**Table 3:** Morphological, physiological, and biochemical characters of the four selected isolates.

Strains	CG3	A79	A111	A93
**Morphological Characters**				
ISP 2-G	++	-	++	++
ISP 2-R	Golden yellow	-	Orange	Narcissus yellow
ISP 2-A	White	-	-	-
ISP 3-G	++	+	++	++
ISP 3-R	Zinc yellow	White	Orange	Colorless
ISP 3-A	White	Cream	-	-
ISP 4-G	++	+	++	-
ISP 4-R	Zinc yellow	Colorless	Orange	-
ISP 4-A	White	-	-	-
ISP 5-G	++	-	++	-
ISP 5-R	Cream	-	Colorless	-
ISP 5-A	White	-	-	-
ISP 6-G	+	-	++	++
ISP 6-R	Golden yellow	-	Honey yellow	Colorless
ISP 6-A	White	-	-	-
ISP 7-G	++	+	++	++
ISP 7-R	Golden yellow	Colorless	Pearlescent copper	Colorless
ISP 7-A	White	Colorless	-	-
**Physiological Characters**				
Temperature range for growth (°C)	25–50 °C/Opt37 °C	25–35 °C/Opt 30 °C	25–35 °C/Opt30 °C	25–35 °C/Opt 30 °C
% NaCl tolerance	(0–18)%/Opt(3–7) %	(0–2.5)%/Opt 0%	(0–2.5)%/Opt 0%	(0–2.5)%/Opt 0%
pH range for growth	6–12/Opt 7	6–9/Opt 7–8	6–9/Opt 7	6–9/Opt 7
**Biochemical characters**				
Glucose (positive control)	+	+	+	+
Arabinose	-	+	+	-
Sucrose	+	++	+	-
Xylose	-	+	+	+
Inositol	+	-	++	+
Mannose	-	++	++	+
Fructose	+	++	+	++
Rhamnose	-	-	+	++
Raffinose	-	-	+	+
Cellulose	-	-	+	-
Urease	+	+	-	-
Gelatin	+	+	+	-

**Morphological characters**: G, growth; R, reverse color; A, aerial mycelium; ++, good growth; +, sparse growth; -, no growth. **Biochemical characters:** -, less than negative control (water); +, similar to positive control (glucose); ++, better than positive control. Opt, optimum.

**Table 4:** Antimicrobial activity of *Actinobacteria* strains isolated from different Algerian regions (mm).

Strains	*K. pneumoniae*	*P. aeruginosa*	*E. coli*	*C. albicans*	*S. aureus*	*M. luteus*	*B. cereus*
C56	-	-	-	-	-	12	12.5
RH94	-	-	-	-	30	28.5	21.5
S29	-	-	-	-	-	9	10
C7	-	-	-	-	-	-	13
V12	16.5	11	13.5	20	19	18	13
V9	-	9	-	11.5	-	-	-
V5	-	-	-	13.5	-	-	-
A28	-	-	-	-	10	-	-
CG3	-	11	30	12	28	30	22
T1	40	-	-	10	30	35	-
BO15	-	-	-	-	12	-	-
S27	-	-	-	-	-	8	-
A23	-	-	13	-	18	19	11
A15	-	-	-	11	-	-	-
A111	-	-	-	-	11	-	-
A79	-	-	12	10	11	-	-

**Table 5:** Antimicrobial activity of the six selected strains (Minimum Inhibitory Concentration: µg/mL).

Isolates	CG3	A79	A111	A15	S27	A23	Positive Control	Negative Control
							**Oxytetracycline**	**Methanol**
*Bacillus subtilis*	0.52	-	-	-	-	-	2.08	-
*Staphylococcus aureus*	2.08	66.66	8.33	-	66.66	4.16	0.05	-
*Micrococcus luteus*	2.08	-	-	-	-	4.16	0.52	-
*Mycobacterium smegmatis*	66.66	-	-	-	-	-	1.66	-
*Chromobacterium violaceum*	1.04	33.33	66.66	-	-	-	0.52	-
*Klebsiella pneumonia*	-	-	-	-	-	-	1.66	-
*Pseudomonas aeruginosa*	66.66	-	-	-	-	-	1.66	-
*Escherichia coli TolC*	2.08	33.33	-	-	-	33.33	1.66	-
							**Nystatin**	**Methanol**
*Mucor hiemalis*	16.66	66.66	-	-	-	-	4.16	-
*Candida albicans*	66.66	-	-	66.66	-	-	8.8	-
*Pichia anomala*	66.66	-	-	-	-	-	4.16	-
